# Microbiota dynamics, metabolic and immune interactions in the cervicovaginal environment and their role in spontaneous preterm birth

**DOI:** 10.3389/fimmu.2023.1306473

**Published:** 2023-12-22

**Authors:** Stanley Onyango, Jia Dai Mi, Angela Koech, Patricia Okiro, Marleen Temmerman, Peter von Dadelszen, Rachel M. Tribe, Geoffrey Omuse, the PRECISE Network

**Affiliations:** ^1^ Department of Pathology, Aga Khan University, Nairobi, Kenya; ^2^ Centre of Excellence Women and Child Health, Aga Khan University, Nairobi, Kenya; ^3^ Faculty of Life Sciences and Medicine, Department of Women and Children’s Health, School of Life Course and Population Sciences, King’s College London, London, United Kingdom

**Keywords:** lactobacilli, preterm births, bacteriocins, lactic acid, pregnancy, probiotics

## Abstract

Differences in the cervicovaginal microbiota are associated with spontaneous preterm birth (sPTB), a significant cause of infant morbidity and mortality. Although establishing a direct causal link between cervicovaginal microbiota and sPTB remains challenging, recent advancements in sequencing technologies have facilitated the identification of microbial markers potentially linked to sPTB. Despite variations in findings, a recurring observation suggests that sPTB is associated with a more diverse and less stable vaginal microbiota across pregnancy trimesters. It is hypothesized that sPTB risk is likely to be modified via an intricate host-microbe interactions rather than due to the presence of a single microbial taxon or broad community state. Nonetheless, lactobacilli dominance is generally associated with term outcomes and contributes to a healthy vaginal environment through the production of lactic acid/maintenance of a low pH that excludes other pathogenic microorganisms. Additionally, the innate immunity of the host and metabolic interactions between cervicovaginal microbiota, such as the production of bacteriocins and the use of proteolytic enzymes, exerts a profound influence on microbial populations, activities, and host immune responses. These interplays collectively impact pregnancy outcomes. This review aims to summarize the complexity of cervicovaginal environment and microbiota dynamics, and associations with bacterial vaginosis and sPTB. There is also consideration on how probiotics may mitigate the risk of sPTB and bacterial vaginosis.

## 1 Introduction

Extensive research has detailed microbiota residing in many human body sites and their contribution to health and disease (1–4). However, within the cervicovaginal despite much research (5, 6) there remains a lack of clinical impact in terms of improving pregnancy outcomes.

Many have reported differences in the cervicovaginal environment in relation to risk of spontaneous preterm birth (sPTB). Mechanistically, a more diverse and inflammatory environment may lead to cervical shortening and an increased risk of microbial invasion of the gravid uterus (7, 8). However, many of the studies of cervicovaginal microbiota are geographically confined, limited in terms of clinical phenotyping, and include study of heterogenous populations; these limitations may have led to overly simplified interpretation of findings. For example, a *Lactobacillus crispatus* dominant environment in pregnancy is often reported as driver of a healthy pregnancy (9–13), with a more diverse environment (associated with the transition to *Lactobacillus iners* dominance) associated with sPTB (11, 14). This may be the case for European dominated populations (15, 16), but it is unclear whether a diverse cervicovaginal microbiota confers a similar risk in other geographically dispersed populations. As such, there is a need to study women from a range of geographical locations and environments, and to develop a deeper understanding of the nuances of bacterial community structure. This is particularly important as no one bacterial species in presence or absence has been consistently associated with poor pregnancy outcomes such as sPTB. The intricate dynamics within a bacterial community, the potential presence of viruses and the distinct metabolic strategies employed by different bacterial species might collectively exert influence over the determination of risk status (13, 17). Such knowledge would provide a greater understanding and context for treatment strategies, such as targeted probiotic interventions. In addition, future studies could integrate knowledge of cervicovaginal environment status with other maternal exposures and deeper phenotyping to acknowledge sPTB as a syndrome, and that the impact of the vaginal microbiota on sPTB risk may be limited to a sub-group of women only.

This review centers on: (1) cervicovaginal microbiota composition and patterns; (2) cervicovaginal microbial metabolites; (3) cervicovaginal bacteria defense mechanisms like bacteriocins, hydrogen peroxide, and biosurfactants in influencing pregnancy outcomes, including sPTB; and (4) effectiveness of interventions such as probiotics for preventing adverse pregnancy outcomes like preterm births.

### 1.1 Cervicovaginal microbiota and environment

The cervicovaginal environment, along with female genital tract as a whole accommodates a microbial biomass, estimated to be 10^10^–10^11^ bacteria for women of reproductive age (18), and comprises many bacterial species (13, 18, 19). The classification of these microbes into five distinct community state types (CSTs) (20–22) has undergone refinement in response to advances in metagenomics sequencing and enhanced representation of samples (23, 24). Four CSTs are dominated by lactobacilli namely; *Lactobacillus crispatus* (CST I), *Lactobacillus gasseri* (CST II), *Lactobacillus iners* (CST III), or *Lactobacillus jensenii* (CST V), while the fifth (CST IV) is characterized by lower proportions of lactic acid-producing bacteria and higher proportions of strict anaerobes (9, 20, 22, 25). Numerous studies have shown the cervicovaginal microbiota of most women is primarily dominated by lactobacilli (20, 24, 26, 27). A study of the vaginal bacterial communities of 396 asymptomatic North American women from diverse ethnic backgrounds (White, Black, Hispanic, and Asian populations) identified a lactobacilli dominance in 73% of women (20). In instances where lactobacilli are not dominant, other lactic acid-producing microorganisms like *Atopobium*, *Megasphaera*, Eggerthella-like bacterium, and/or *Leptotrichia* species may establish colonization (27, 28). These opportunistic pathogens have demonstrated distinct associations with conditions like bacterial vaginosis (BV), aerobic vaginosis (AV), and sPTB (12, 29–31), although their mere presence or absence is insufficient to explain causality. In a sub-optimal cervicovaginal microbiota environment, lactobacilli may lose their protective capacity to increased inflammation from opportunistic pathogens (32). Certain key genera, including members of *Gardnerella* clades, *Atopobium*, *Prevotella*, *Peptostreptococcus*, *Mobiluncus*, *Sneathia*, *Leptotrichia*, *Megasphera spp*, mycoplasma, have been identified as potential causative agents of sPTB (12, 33).

**Figure 1:**
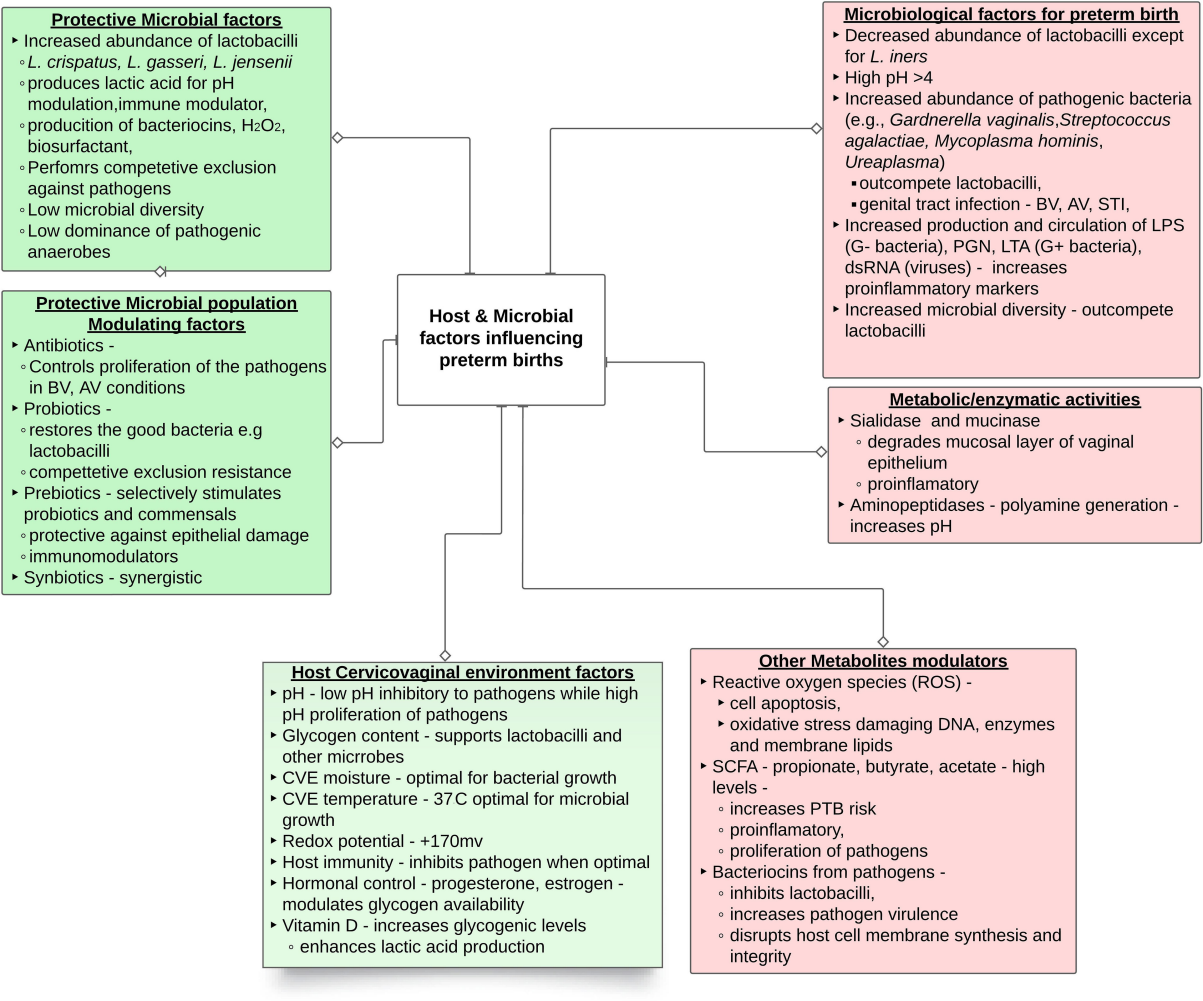
Summary of female genital microbiological factors influencing preterm birth. The abbreviations are dsRNA, double-stranded RNA; LPS, lipopolysaccharide; LTA, lipoteichoic acid; PGN, peptidoglycan; CVE – cervicovaginal environment; SCFA short chain fatty acid.

The cervicovaginal microbial environment is a summative reflection of microbial community and host interactions where several factors tend to influence the cervicovaginal microbiota and, by extension, sPTB (**Figure 1**). Among these is the cervicovaginal pH which is usually maintained in an acidic pH range (3.8 - 4.5) by microbial (85%) and cervicovaginal epithelial (15% [L-lactic acid]) production of lactic acid (34, 35). The interplay between cervicovaginal microbiota and other host environmental factors such as warm, humid, anaerobic, and nutrient-rich cervicovaginal environment, significantly modulates the growth and survival of the cervicovaginal microbiota (18). Cervicovaginal microbiota can equally be influenced by factors related to the host innate immunity (e.g., antimicrobial peptides, cytokines, neutrophils, and monocytes) (36), as well as conditions like diabetes (37), autoimmune disease (38), hormonal fluctuations (39, 40), age (41), sexual activity (42) and the use of antibiotics, the effects of which have been extensively documented (43). Any disruption to the ‘normal’ cervicovaginal microbiota can stimulate the overgrowth of opportunistic pathogens. This overgrowth can outweigh the beneficial microbiota, subsequently increasing the risk of infection (e.g. BV) and sPTB (31, 33).

The menstrual cycle represents a significant influencing factor on the cervicovaginal microbiota (44, 45). Notably, the alkaline pH of the cervicovaginal environment during menstruation, has been linked to the promotion of suboptimal microbiota composition in most women (46). It has been observed that women with a predisposition to pregnancy failure, including those with recurrent miscarriage and recurrent implantation failure, consistently exhibit a reduced abundance of lactobacilli throughout all phases of the ovulation cycle (46). This phenomenon may contribute to increased bacterial diversity, particularly in conditions such as AV and BV which have been associated with increased sPTB risk (31, 33).

While extensive research has been directed towards bacteria, the cervicovaginal microbiota encompasses a dynamic and intricate ecosystem including protozoa, archaea, viruses, and fungi (47). Among these, viruses, including human immunodeficiency virus (HIV), herpes simplex virus-2, and human papilloma virus (HPV), appear to contribute to PTB (47–49); while the role of other viruses remains unclear. Similarly, most fungi, across genera such as *Candida*, *Cladosporium, Pichia, Aspergillus*, and *Rhodotorula* predominantly inhabit the cervicovaginal and may be pathogenic (50, 51). Evidently, further research is essential to comprehend the roles of protozoa, viruses, fungi, and archaea within the cervicovaginal microbiota. The ensuing sections of this review will focus predominantly on bacteria, to shed light on the intricate mechanisms underpinning interactions, shifts, and potential disruptions within cervicovaginal environment during pregnancy and their consequential effects on sPTB, as diagrammatically represented in **Figure 1**.

### 1.2 Cervicovaginal microbiota dynamics during pregnancy

The cervicovaginal microbial community is dynamic and undergoes significant changes during pregnancy (9, 52). These shifts, a result of microbial competition, along with hormonal, immunological, and metabolic interactions, hold potential to ultimately impact pregnancy outcomes. Women with normal pregnancies tend to exhibit a higher dominance of lactobacilli in their cervicovaginal microbiota compared to non-pregnant women (53). A retrospective longitudinal case-control study demonstrated a higher abundance of *Limosilactobacillus vaginalis*, *L. crispatus*, *L. gasseri*, and *L. jensenii* and a lower abundance of 22 other phylotypes in pregnant women (9). The microbiota composition, particularly the presence of lactobacilli, exhibited greater stability in pregnant women when compared to women who were not pregnant (9). This may explain the connection between lactobacilli and full-term pregnancy.

Within a predominantly African-American cohort, (n=474 women), longitudinal characterization of vaginal microbiota during pregnancy determined a decrease in richness and evenness of the vaginal microbiota from first through the third term of gestation (24). In addition, advancing gestational age corresponded with lactobacilli dominance, including a positive correlation with over 30 lactobacilli species and a negative correlation with opportunistic pathogens (24). This correlation highlights the critical role of lactobacilli in promoting term birth. However, a small study of 12 women identified a stable cervicovaginal microbiota, with no changes in lactobacilli (particularly *L. crispatus and L. iners*) observed before and during pregnancy (54).

Across trimesters, a lactobacilli-dominant microbiota by the second trimester has been proposed to be a marker for a stable environment conducive to term birth, irrespective of whether the starting cervicovaginal microbiota was simple or complex (55). DiGiulio and colleagues demonstrated a stable vaginal community state on the time scale of weeks but noted that some women maintained a stable CST throughout pregnancy, whereas others had relatively frequent transitions between CSTs. However, these shifts did not influence term and sPTB (25). The study further discovered that CSTs dominated by lactobacilli exhibited greater stability compared with those with frequent transitions. Moreover, both the duration and proportion of time spent in frequent transition were correlated with sPTB (25). These findings highlight the intricate interplay of vaginal microbiota without a direct correlation to delivery outcomes, prompting further exploration into the multifaceted factors that influence pregnancy and childbirth. In another longitudinal study, even though its primary focus was not on sPTB, the cervicovaginal microbiota of 57 women was sequenced at 24, 36 weeks of gestation and at birth. With advancing gestational age, the study unveiled an increase in the relative abundance of diverse bacterial taxa. A gradual decline in lactobacilli abundance which coincided with an increase in *Enterococcus*, *Streptococcus*, and bacteria within the Enterobacteriaceae family was also reported (56). These bacteria, commonly defines AV and has also been linked to sPTB (57).

Term birth has consistently been correlated with an abundance of lactobacilli, mainly due to their lactic acid production. This lactic acid plays a crucial role in maintaining an acidic pH within the cervicovaginal environment, promoting the growth of beneficial lactobacilli while inhibiting opportunistic pathogens (9). In the cervicovaginal environment, lactobacilli are the primary producers of lactic acid, which exists in two forms: L-type and D-type (58) from glycogen and its hydrolysates (59, 60). The former is produced by both cervicovaginal epithelial cells and bacteria, while the latter is predominantly bacterial-derived, accounting for over 85% of cervicovaginal lactic acid (35). Among the lactobacilli species, *L. crispatus* and *L. gasseri* are prominent in cervicovaginal metabolism, capable of producing both D and L-type lactic acid, while *L. jensenii* exclusively produces the D-type, and *L. iners* generates the L-type (60), emphasizing the influence of predominant lactobacilli species on lactic acid composition and the unique metabolic profile of the cervicovaginal environment. This intricate interplay between lactobacilli and lactic acid has wider implications for pregnancy outcomes. Elevated levels of L-lactic acid and the L:D-lactic acid ratio stimulate extracellular matrix metalloproteinase inducer (EMMPRIN, also known as CD147) and matrix metalloproteinase MMP-8 (60). EMMPRIN, a crucial protein in fetal development, modulates MMP-8 expression (61). MMP-8, functioning as an enzyme, degrades the extracellular matrix of cervicovaginal epithelial cells (60), potentially compromising cervical integrity and facilitating bacterial movement, thereby increasing the risk of sPTB (60). In cervicovaginal environments with lower lactobacilli dominance but an abundance of *L. iners*, L-lactic acid levels tend to be higher (60). This observation provides valuable insights into the relationship between *L. iners* and sPTB.

#### 1.2.1 Hormonal influence on cervicovaginal microbiota and pregnancy

Throughout pregnancy up to the delivery of the placenta, the concentrations of progesterone and estrogen continue to rise [Farage, Miller, & Sobel, (62)]. Lactobacilli dominance in the cervicovaginal environment, influenced by estradiol and/or progesterone, is prevalent during reproductive years. However, in early stage of puberty and post menopause women often exhibit a more diverse microbiota with lactobacilli deficiency [Kaur et al., 45; Muhleisen & Herbst-Kralovetz, (63)] correlating with an increased risk of sPTB. Elevated estrogen levels stimulate glycolytic and lactic acid fermentation which play a crucial role in fostering lactobacilli growth and normal microbiota [Farage et al., (62); Kim & Park, (64)]. Conversely, in postmenopausal women, reduced estrogen secretion leads to a decline in protective lactobacilli such as *L. crispatus*, resulting in microbiota imbalance and increased colonization by microorganisms like *G. vaginalis* and *Candida* [Gustafsson et al., (65); Kim & Park, (64)].

The delicate balance between progesterone and estrogen is paramount. Progesterone withdrawal triggers increased expression of estrogen-dependent receptors, including oxytocin, gap junction protein connexin 43 (CX-43), cyclooxygenase-2 (COX-leading to prostaglandin production), prostaglandin, and myosin light chain kinase (MLCK) receptors (66). This cascade promotes uterine contractility, emerging as a potential risk factor for sPTB. Notably, vitamin D, with progesterone-like activity, is implicated in maintaining and reinforcing the cervicovaginal epithelium through the induction of the antimicrobial peptide LL-37 (67). Additionally, vitamin D regulates insulin synthesis, a crucial hormone for glycogen metabolism. Vitamin D deficiency during pregnancy is associated with an increased risk of sPTB due to its impact on glycogen metabolism (67, 68).

### 1.3 Preterm birth and infection

Preterm birth (PTB), defined as childbirth occurring before 37 weeks of gestation, constitutes approximately 10-15% of global births (69, 70). PTB contributes significantly to neonatal morbidity and mortality, and its consequences can extend into adulthood (71). Approximately one-third of PTBs are medically indicated, often due to maternal or fetal conditions like preeclampsia or growth restriction, while the remaining are spontaneous PTBs, with 25-30% involving spontaneous preterm labor, with or without prelabor rupture of fetal membranes (PPROM) (72).

Our primary focus is how cervicovaginal microbiota modulate the risk of sPTB, recognizing that sPTB is a complex syndrome influenced by multiple factors, including intra-amniotic infections, ascending infections, cervical insufficiency, stress, vascular disorders, maternal age, multiple pregnancies, nutrition, immunity, and lifestyle behaviors (72–74). Cervicovaginal infections during pregnancy often manifest as polymicrobial rather than single bacterial species. About 35% of sPTB cases are associated with infections and inflammation (75). Key microorganisms in this context include *C. trachomatis*, *Enterococcus* spp., *Gardnerella vaginalis (G. vaginalis)*, *Mycoplasma hominis*, *Ureaplasma parvum (U. parvum)*, and *Ureaplasma urealyticum (U. urealyticum)*, which can significantly influence cervical health and pregnancy outcomes (12, 57, 76–79). Shedding light on this intricate relationship, a study conducted in pregnant Caucasian women identified a microbial community (CST IV) characterized by an enrichment of facultative anaerobes and a low abundance of lactobacilli (80). This specific microbial composition has been associated with both cervical shortening and an elevated risk of sPTB (80). In this context, *U. urealyticum* emerges as the commonly isolated microorganism in the cervix of patients experiencing PTB, with a prevalence estimated at 34.5%, followed closely by *Enterococcus* spp. at 27.6% and *Mycoplasma hominis* at 17.2%. Additionally, PCR analysis has identified *U. parvum* as the dominant pathogen in the cervix, accounting for 85.5%, followed by *Chlamydia trachomatis* (*C. trachomatis*) at 8.0% (81), confirming the importance of *Ureaplasma* spp. in endocervical infection and its association with placental inflammation (82), pPROM and sPTB (83). Similarly, the prevalence of *C. trachomatis* has been observed to increase among women experiencing preterm labor, particularly those with PTB, compared with women who gave birth at term (84). This became apparent when women with *C. trachomatis* receiving antibiotic treatment showed a reduced incidence of sPTB (85). However, there is some inconsistency regarding the impact of *C. trachomatis* on sPTB. In a case-control study involving 75 Iranian women with sPTB, no significant association with *C. trachomatis* cervical infection was observed when compared with 75 women with term births (86). Nevertheless, women with cervical *C. trachomatis* infection who received antibiotic treatment had lower rates of pPROM, sPTB, and small-for-gestational-age infants compared with those who did not receive antibiotic treatment (87, 88), providing insight into the role of *C. trachomatis* in promoting sPTB.

*G. vaginalis* is another important cervical microorganism, and its abundance has been linked with microbial invasion of the amniotic cavity, consequently promoting pPROM (89). Interestingly, a case-control study involving 203 pregnant Korean women in mid-pregnancy revealed a contradictory finding, associating *G. vaginalis* with term birth (90). This study also highlighted a positive correlation between *Ureaplasma* and *Prevotella* and sPTB but only in the absence of lactobacilli (90); highlighting the need to examine the cervicovaginal microbiota as a community.

Moreover, there is a significant variation in the risk of sPTB linked to cervicovaginal infections at different times during pregnancy. In the second trimester, when the cervix is typically stable, such infections do not significantly raise the sPTB risk. However, infections in the third trimester, when the cervix naturally prepares for labor, show a marked increase in the risk, especially when they coincide with premature cervical ripening (91). This temporal distinction underscores the critical importance of considering pregnancy stage when assessing the impact of cervicovaginal infections on sPTB risk.

### 1.4 Cervicovaginal microbiota, race, and ethnicity, as sPTB risk factors

As stated, elevated risk of sPTB has been associated with specific microbiota compositions in the cervicovaginal, including reduced abundance of *L. crispatus* and increased dominance of *L. iners* in European and American populations (6, 15, 16). Recent studies have revealed distinct cervicovaginal microbiota based on geographical locations, emphasizing the importance of considering geographical diversity in cervicovaginal microbiota research to better comprehend sPTB risk factors. When comparing the cervicovaginal microbiota profiles of African American and Caucasian women, 30-60% of African-American, African, and Hispanic women are more likely to possess diverse cervicovaginal microbial communities with low lactobacilli dominance, increasing their risk of sPTB (15, 31, 92). This knowledge gap is significant because 80% of sPTB occur in developing countries, possibly due to the high prevalence of vaginal infections like AV and BV, coupled with inadequate healthcare facilities, as reported among sub-Saharan African women (93).

The influence of cervicovaginal microbiota on pregnancy outcomes in African women remains unclear. The cervicovaginal environment of approximately 16% of pregnant women in Pemba Island, Tanzania, are colonized by at least three of four STI (sexually transmitted infection) infectious agents (i.e., *Chlamydia trachomatis*, *Trichomonas vaginalis*, *Neisseria gonorrhoeae*, and *Mycoplasma genitalium*); therefore, adverse pregnancy outcomes may not necessarily be instigated by a single infectious agent (79). In Kenya, more than fifty percent of the vaginal microbiota consisted of *Gardnerella* and/or *Prevotella*, with lactobacilli ranging from 1% – 99%, but without a clear link between variation in composition and term or sPTB (94). In a longitudinal study involving pregnant Nigerian women (n=38), the prevalent cervicovaginal bacterium was *L. iners* (40). Noteworthy, this prevalence of *L. iners* was particularly pronounced in most women who ultimately delivered at full term, challenging a direct association between *L. iners* and sPTB (40). The presence of *L. iners* in the cervicovaginal environment indicates it is a regular part of a healthy vaginal microbiota.

### 1.5 Cervicovaginal metabolic interaction and link with sPTB

It is important to note that the causality of sPTB cannot be solely attributed to the presence or absence of specific bacteria. The composition of the cervicovaginal microbiota and the diversity of microbial species within it, plays a significant role in shaping the metabolite profile within the cervicovaginal environment (13, 60, 95) owing to the inherent metabolic capability of microbiota (96). These metabolites can influence cervicovaginal homeostasis (60, 95), yet the precise mechanisms by which their interaction impacts sPTB remain elusive. The dynamic metabolic ecosystem within the cervicovaginal environment encompasses diverse pathways involving carbohydrates and amino acids, contributing to its complex nature, believed to influence pregnancy outcomes. For example, the metabolomes of women who experienced sPTB and those who carried their pregnancies to term differ in 30 of 310 (9.7%) identified metabolites. Notably, mannitol and methylphosphate were upregulated, while 28 other metabolites, including medium-chain fatty acids and collagen degradation markers, were downregulated in women who had sPTB compared with term births (97). In a separate investigation involving 300 healthy pregnant women, women with a higher presence of *L. jensenii* CST V, as opposed to *L. crispatus* CST I (both considered health-promoting bacteria), in their vaginal fluid exhibited lower levels of lactate and glutamate. This subgroup, characterized by low glutamate and D-lactate levels and high acetate levels, was notably more prone to sPTB compared to those dominated by *L. crispatus* CST I, as confirmed by both a study involving this subgroup (98) and a prospective study with asymptomatic singleton women (99). Furthermore, the composition of metabolites in the cervicovaginal space changes over gestation; at 20-24 weeks and 24-28 weeks there are significant differences in 313 amino acids, carbohydrates, and peptide metabolites between women experiencing sPTB and term birth (100). However, the number of distinct metabolites reduced from 82 at 20-24 weeks to 48 at 24-28 weeks (100).

Some studies, like Melinda et al. [91], did not find significant metabolite differences between term birth and sPTB, potentially due to early sample collection. However, Gerson et al. [92] identified 11 metabolites associated with sPTB, including stearoyl-linoleoyl-glycerol, palmitoyl dihydrosphingomyelin, and others. Amino acid catabolites (biogenic amines) were increased in women with CST IV microbiota profiles, while asparagine was reduced compared to CST I profiles. These findings were corroborated in another study, where women with CST IV had elevated amino acid catabolites and 12-HETE [93]. In contrast, levels of certain amino acids, specifically glutamate and tyrosine, as well as dipeptides, were found to be low in this context, with implications for signaling cascades for host inflammation and disruption of epithelial barrier integrity; collectively these may contribute to the occurrence of sPTB (101). Another study involving 346 pregnant women examined during the first two trimesters, including 60 cases of sPTB, did not reveal disparities in metabolite profiles between women who experienced sPTB and those who carried their pregnancies to term (13). Nonetheless, the authors identify metabolite features that increased in cases of sPTB such as betaine, acetate, and calcium levels, whereas lactate and leucine were positively correlated with full-term births (13). Interestingly, aspartate seemed to have a protective effect in the presence of *L. acidophilus*, but increased the risk of sPTB when *Bifidobacterium breve* and *L. delbrueckii* were present (13).

While limited studies have explored metabolic relationships among microbes in the cervicovaginal environment concerning sPTB, a significant *in vitro* study by Horrocks et al. revealed a symbiotic and commensal relationship among bacteria commonly associated with BV infection and sPTB (102). Notably, *Prevotella* spp., such as *P. bivia*, a group of bacteria frequently linked to BV infections and sPTB, exhibited distinctive symbiotic connections with *Peptostreptococcus anaerobius* and *G. vaginalis* (102). *P. bivia* plays a pivotal role in this relationship by significantly releasing metabolites actively utilized by other BV-associated bacteria. These metabolites include succinate, fumarate, alanine, glutamate, glycine, methionine, phenylalanine, proline, valine, and uracil, highlighting the crucial role of *P. bivia* in cross-feeding within the cervicovaginal environment. Furthermore, *P. bivia* and *P. anaerobius* maintain a commensal relationship within cervicovaginal environment where *P. bivia* provides amino acids (threonine, tyrosine, phenylalanine, and proline), especially proline, to *P. anaerobius*, promoting increased glucose uptake and acetate production from glucose (102). It is thought that *G. vaginalis*, by aiding the ascension of *P. bivia*, enhances its invasive potential in the uterus (103). Additionally, *P. bivia* and *G. vaginalis* exhibit a mutualistic relationship, leading to increased production of acetate and aspartate, which has been linked to BV and sPTB (13, 99, 102). *G. vaginalis* closely associates with *L. iners* and less with other lactobacilli species, including *L. crispatus*, a profile known to be correlated with BV and sPTB (11, 13). Interestingly, *L. iners* is unable to produce acetate, suggesting that acetate production originates from other bacteria, including *G. vaginalis* (102). Therefore, it remains unclear how *L. iners* and *G. vaginalis* interact to promote sPTB.

Despite certain limitations in study design (e.g., non-representative media, microbiota, and host immunity), it is important to consider the diversity of metabolic relationships within and between bacterial species, rather than relying solely on ecological consistency based on marker genes in BV infection and sPTB.

### 1.6 Cervicovaginal microbiota instigated immune modulation on sPTB

The cervicovaginal microbiota significantly influences host immunity pregnancy outcomes, with interactions implicated in intrauterine infections (104). Microbial species, such as Enterobacteriaceae *spp*, *Streptococcus spp*, *Staphylococcus spp*, *Escherichia coli*, and Gram‐negative bacteria, may migrate through anatomical barriers, such as cervicovaginal or endometrial epithelia, triggering elevated production of microbial products like lipopolysaccharides and inflammasomes, altering cervicovaginal inflammatory responses (105). This heightened immune response, involves the activation of immune components such as the complement system (C3b and C5), mannose-binding lectin (MBL), immunoglobulins (IgM and IgG), alongside the production of pro-inflammatory cytokines (IL-8, IL-6, and IL-1β) (36). Immune alterations, particularly the association between the dominance of *L. iners* and elevated levels of IgM, C3b, C5, C5a, and IL-6, collectively contribute to an increased susceptibility to short cervix, compromised endometrial receptivity, implantation rates and increased susceptibility to sPTB (36, 105). *Prevotella* and *Gardnerella*, integral to BV, have also been shown to mediate cervicovaginal epithelium immune response. They have been demonstrated to activate NF-κB, induce the release of proinflammatory cytokines, including TNF-α, particularly through TLR2/4 pathways (106, 107), and elicit the NLRP3 inflammasome release via NOD-like receptors (NLRs) signaling pathways (108). This dual influence on TLR2/4 and NLRs expression apart from contributing to cervical inflammation, may also increase the risk of sPTB.

Neutrophils, which are involved in cervical remodeling can infiltrate the myometrium, potentially leading to cervical shortening during labor, to equally increase the susceptibility to sPTB (109, 110). From limited data, it appears that neutrophil counts in cervicovaginal fluid decrease as gestational age increases (111). Notably, women with a low diversity cervicovaginal microbiota, characterized by the dominance of *L. iners* (CST III), tend to exhibit an increased presence of neutrophils in the cervicovaginal environment during pregnancy (111, 112). While one study revealed a positive correlation between *G. vaginalis* (CST IV) and neutrophils, suggesting increased neutrophil presence in samples from women who eventually experienced sPTB (111), a study by Molina et al. study provided contrasting results (112). These conflicting results underscore the complexity of the relationship between the cervicovaginal microbiota and the neutrophil response during pregnancy.

Other than the presence or absence of key microbial species, cervicovaginal microbiota can also modulate immunity and pregnancy outcome via the production of lactic and short-chain fatty acids (SCFAs). Lactic acid can exert immune suppressive effects by inhibiting monocyte and cytotoxic CD8+ T cell differentiation, dendritic maturation, and macrophage polarization towards the M2 type (113). This potentially maintains the integrity of the cervix to promote term birth. Reduced lactic acid levels, often associated with a low presence of lactobacilli, can further contribute to elevated levels of inflammatory immune factors such as IL-6, IL-8, IL-1α, IL-1β, and MIP-1α/β (32) which can together compromise the cervix, potentially enabling sPTB.

Although SCFAs are recognized for their beneficial effects on the gut such as enhancing the integrity of the gut epithelial membrane (114), their impact on the cervicovaginal environment appears to be detrimental. SCFAs are organic fatty acids typically containing fewer than six carbon atoms. Common SCFAs include acetic acid (C=2), propionic acid (C=3), and butyric acid (C=4) (115). Elevated levels of SCFAs, such as propionate, succinate, acetate, and butyrate, are linked to increased inflammatory markers including IL-1β, IL-2, IL-6, IL-8, IL-10, TNF-α, Interferon (IFN)-γ, and RANTES (59, 116). Some of these inflammatory markers, for example acetate in early pregnancy, have been associated with sPTB in cases where the cervicovaginal microbiota is less dominated by *L. crispatus* (13). Furthermore, high SCFA levels can dampen the expression of an innate antibacterial factor known as neutrophil gelatinase-associated lipocalin (NGAL), a condition that can promote the growth of the BV community (117) to promote sPTB. In cases of BV, a high concentrations of acetic (100 mM) and butyric (20 mM) acids can specifically stimulate TLR1/2/3 in cervicovaginal epithelial cells, leading to increased secretion of the TNF-α while inhibiting the production of IL-6, RANTES, and IP-10 (34). Despite the observed effects, the exact impact of cervicovaginal microbiota on the immune responses of reproductive cells and its consequences on spontaneous preterm birth (sPTB) are not yet clear and require further clarification.

### 1.7 Other metabolites – hydrogen peroxide, bacteriocins, biosurfactant, sialic acid and sialidase activity

Beyond lactic acid and SCFAs, beneficial bacteria produce additional metabolites such as hydrogen peroxide, bacteriocins, and biosurfactants, all of which contribute substantially to maintain the health and homeostasis of the cervicovaginal environment (95).

#### 1.7.1 Hydrogen peroxide

Hydrogen peroxide (H_2_O_2_) is a reactive oxygen species that is produced by specific lactic acid bacteria such as *L. crispatus* and *L. jensenii, L. gasseri, and L. vaginalis* (118) but not *L. iners* (119). The extent of H_2_O_2_ production varies among these bacteria, with higher production being observed with *L. jensenii* and *L. vaginalis* (118). Mechanistically, H_2_O_2_ can inhibit the growth of pathogenic microbes such as *G. vaginalis* and *C. albicans* by the oxidation of sulfhydryl groups in proteins and essential enzymes, DNA, and other cellular components (120). This reduction in viability hinders the ability of these opportunistic pathogens to adhere to the vaginal epithelium and cause ascending uterine infections (118). Moreover, the presence of H_2_O_2_-producing lactobacilli can also regulate the immune response by suppressing proinflammatory cytokines including interleukin-1β (121).

#### 1.7.2 Microbiota bacteriocins as an agent of cervicovaginal microbiota modulation and sPTB

Bacteriocins as summarized in **Table 1**, are small peptides produced in favorable conditions by some cervicovaginal microbes and can kill or inhibit the growth of opportunistic pathogens. Examples include compounds such as crispacin produced by *L. crispatus* along with lactocin 160 and lactocin AL705 (122). Some disrupt the integrity of the target bacterial cell membrane, causing cell death, while others interfere with essential metabolic pathways, such as DNA, RNA, and protein metabolism inside the cell, thereby killing and/or inhibiting microbial growth (135). They are effective against several pathogenic bacteria, including *G. vaginalis* and *Streptococcus agalactiae*, which have closely been associated with BV and sPTB respectively (136). *L. rhamnosus* may significantly contribute to the reduction of sPTB risk posed by *P. bivia* and *G. vaginalis*. It can achieve this by producing lactocin 160 and AL705, which target the cytoplasmic membrane of these bacteria, leading to the efflux of ATP molecules and the dissipation of the proton motive force (132).

**Table 1:** A summary of bacteriocins produced by bacteria from the female genital tract, their molecular weight, and mode of action.

Vaginal Bacteria	Bacteriocin	Molecular Weight	Properties	Mode of Action
*Lactobacillus crispatus*	Crispacin A	5.4 kDa (122)	Stable at pH 2-10, heat stable	Pore-forming disrupts the cell membrane
*Lactobacillus gasseri*	Gassericin A	3.8 kDa (123)	Heat stable	Pore-forming disrupts the cell membrane
*Limosilactobacillus fermentum*	Fermentcin B	3-5 kDa (124)	Heat and stable pH (3.0-8.0)	Binds to lipid II, inhibits cell wall synthesis
*Gardnerella vaginalis*	Vaginolysin	57 kDa (125)	Hemolytic, immunosuppressive	Pore-forming disrupts the cell membrane
*Enterococcus faecalis*	Enterocin II	5-10 kDa (126)	Heat-stable, acid and alkali tolerant	Inhibits cell wall synthesis and causes membrane depolarization
*Streptococcus anginosus*	Angicin	<10 kDa (127)	Heat and acid stable	Membrane permeabilization
*Bifidobacterium bifidum*	Bifidocin B	3.3 kDa (128)	Heat and acid stable	Membrane permeabilization
*Escherichia coli*	Colicin E1	57 kDa (129)	Heat stable, pH stable	Forms a pore on the cytoplasmic membrane leading to membrane depolarization and cell death
*Staphylococcus aureus*	Aureocin A53	6.0 kDa (130)	Heat and acid stable	Pore-forming disrupts the cell membrane
*Limosilactobacillus reuteri*	Reutericin 6	2.7 kDa (131)	Heat stable	Disrupts cell membrane
*Lacticaseibacillus rhamnosus*	Lactocin 160	3.8 kDa (132)	Heat stable	Causes an efflux of ATP and dissipation of the proton motive force
*Ligilactobacillus salivarius*	Salivaricin	4.8 kDa (133)	Heat, pH stable	Causes an efflux of ATP and dissipation of the proton motive force
*Lactobacillus iners*	Inecin L	3.2 kDa (134)	Not known	Inhibits the cell wall biosynthesis.

These bacteriocins are important in modulating microbiota and host immunity.

It is important to note that the production of bacteriocins is not exclusive to beneficial bacteria. Pathogenic bacteria can similarly produce bacteriocins to enhance their pathogenicity against the host and other resident bacteria. For example, *L. iners* and *G. vaginalis* produce cytolysins, namely inerolysin (125, 137) and vaginolysin (137) respectively. These can promote cervicovaginal epithelial damage to increase inflammation to increase the risk of sPTB. The role of bacteriocins remains incompletely understood. However, the type and rate of bacteriocin production, whether from pathogenic or commensal bacteria, is heavily influenced by the conditions within the cervicovaginal environment (125, 137). Further investigations are essential to characterize properties and functions of bacteriocins, to increase our understanding of their role in either preventing or promoting adverse pregnancy outcomes and their broader implications for human health.

#### 1.7.3 Cervicovaginal Biosurfactants and link to sPTB

Biosurfactants are another category of molecules produced by cervicovaginal microbiota and are surface-active molecules generated by microorganisms. These compounds can either reside on the cell surface or be secreted extracellularly. Biosurfactants serve varying functions, microbial quorum sensing, motility, biofilm adhesion and detachment, virulence factors, and antagonism (138). Beneficial bacteria can use biosurfactants to reduce the adhesion of pathogenic bacteria to the cervicovaginal epithelium, thereby discouraging the adherence of pathogenic bacteria (139) to maintain balanced cervicovaginal microbiota promote term birth. Certain bacteria can use biosurfactants to establish a biofilm, adhering to the cervicovaginal surfaces for growth and survival (139). Furthermore, pathogenic bacteria within the biofilm are challenging to control due to the protective layers they form against host defenses and antibiotics (39, 139). This environment may also enhance the survival and growth of opportunistic pathogens capable of initiating sPTB.

#### 1.7.4 Cervicovaginal microbiota and sialic acid on sPTB

Elevated sialidase enzyme activity, which catalyzes the production of sialic acid, has been associated with various adverse pregnancy outcomes, including placental infection, miscarriage, late pregnancy sPTB, and recurrent BV (140, 141). Sialic acid is a sugar molecule located on the terminal residues of glycoproteins and glycolipids present on cell surfaces, including the cervicovaginal epithelial cells (141). Certain bacteria, including *G. vaginalis* and *A. vaginae*, secrete sialidase and other mucin-degrading enzymes (142–144) to produce sialic acid. By degrading the terminal sialic acid residues from the protective glycan mucus layer of the cervicovaginal epithelium, these bacteria can trigger inflammation and disrupt the balance of the cervicovaginal microbiota which is associated with sPTB and low birth weight (142). The degradation of the mucus also generates readily available carbon sources that can contribute to the growth of other bacteria within the cervicovaginal environment (144). In a pregnant Chinese cohort (n=819) high sialidase activity and leukocyte levels were linked to diverse vaginal microbiota composition and reduced lactobacilli; however, these factors did not increase the risk of PTB (145). The variation in results could be influenced by other factors, such as ethnicity, which is known to play a significant role in shaping the composition of vaginal microbiota and its effects on pregnancy outcomes.

### 1.8 Probiotic-based interventions in modulating the cervicovaginal-microbiota-PTB link

The modulation of cervicovaginal microbiota can be achieved through interventions, including the use of probiotics, prebiotics, antibiotics, and pH modulators (146). Perhaps the most common and effective method that has been employed against BV, AV, and associated infectious conditions, is the use of antibiotics. Antibiotics such as metronidazole, clindamycin, and azithromycin are commonly used (43, 77, 147). Antibiotics, while effective against infections, can disrupt the microbiota balance, including beneficial bacteria. This disruption is associated with a higher recurrence rate (43) and an increased risk of sPTB (147).

In response, alternatives to antibiotics such as probiotics have been proposed. Probiotics encompass live beneficial bacteria that can colonize the vagina and improve its microbial composition. For example*, L. crispatus, L. jensenii*, and *L. gasseri* are commonly used probiotics (147, 148). They can be administered orally or intravaginally in the form of capsules, suppositories, or creams (146, 149–151).

Most interventions have focused on using *Lactobacillus* species to address bacterial conditions such as BV and AV, both of which have been associated with an increased risk of sPTB. The results of these interventions have yielded mixed findings, with some studies demonstrating the effectiveness of probiotics while others have not. For instance, in a study involving pregnant women who received vaginal supplementation with *Lactobacillus crispatus* CTV-05, a reduced rate of sPTB was observed (148). Another study involving pregnant women who tested positive for Group B *Streptococcus* (GBS) during late pregnancy administered a daily oral probiotic capsule containing *L. rhamnosus* GR-1 and *L. reuteri* RC-14 until delivery. This intervention resulted in a modest reduction in GBS compared to the placebo group (**Table 2**) (153). Additionally, although not conducted in pregnant women, a randomized controlled study found that vaginally applied probiotic capsules containing *L. rhamnosus* GR-1 and *L. reuteri* RC-14, once a day for five days, led to a faster BV cure rate compared to twice-daily metronidazole gel application (150). Some studies have also shown that these interventions can reduce inflammation, which is a known risk factor for sPTB. In one study, a capsule containing *L. brevis* CD2, *L. salivarius* subsp. *salicinius*, and *L. plantarum* was administered vaginally to pregnant women with BV for 30 days, proving effective against BV and reducing vaginal inflammatory cytokines: IL-1β and IL-6 (151).

**Table 2:** A summary of probiotics, prebiotics and synbiotic intervention to influence female genital tract microbiota and consequence on bacterial vaginosis, anaerobic vaginosis, and or pregnancy outcome.

Subjects	Intervention	Region	Outcome	Reference
140 Pregnant BV/sexually active	RDBPCT - vaginal 10^9^ cfu each of *L*. *brevis* CD2. *L*. *salivarius* subsp. *salicinius*; *L*. *plantarum* 1 capsule/o.d./30d	India	-Probiotics prevented BV better than pH tablets in healthy subjects -Lactobacilli reduced IL-1*β* and IL-6 vaginal cytokines -Lactobacilli-containing tablets can cure BV and reduce vaginal inflammation	(151)
66 pregnant (32 treatment group, 34 placebo) with BV	RDBPCT - oral 2.5 × 10^9^ cfu each of *L. rhamnosus* GR-1 *L. reuteri* RC-14 1 capsules/t.d./84d	Finland	-no significant difference in Shannon diversity between placebo and probiotic group -flux irrespective of the treatment	(152)
61 pregnant	vaginal 2 x 10 ^9^ cfu each of *Lactobacillus crispatus* CTV-05 1 capsule/o.d./5 d then once weekly for 6 weeks	UK	-reduced rate of early PTB <34 weeks	(148)
99 (50 placebo/49 probiotic) pregnant in 35-37 weeks with Group B Streptococcus (GBS)-positive	RDBPCT - oral 1 x 10 ^9^ cfu each of *L. rhamnosus GR-1 and L. reuteri RC-14*, 2 capsule and placebo/o.d./until delivery	China	-42.9% of pregnant probiotic group turned negative. -18% placebo group turned negative	(153)
238 (115 placebo/123 probiotic) pregnant in 9-14 weeks	RDBPCT – oral 2.5 x 10 ^9^ cfu each of *L. rhamnosus GR-1 and L. reuteri RC-14*, 2 capsule and placebo/o.d./until delivery	UK	-rate of BV in the probiotic group was 15% v 9% placebo no difference in probiotic colonization no significant difference in alpha diversity	(154)
271 (136 placebo/135 probiotic) pregnant in <12 weeks	RDBPCT - oral 1 x 10 ^9^ cfu each of *L. rhamnosus GR-1 and L. reuteri RC-14*, 1 capsule and placebo/o.d./8weeks	Germany	-proportion of normal vaginal microbiota reduced in both groups	(155)
34 female subjects (aged between 18 and 50) with BV	RPCT - vaginal *L. fermentum* LF15 (DSM 26955) *L. plantarum* LP01 (LMG P-21021) 4 × 10^8^ cfu/dose 1 capsule/o.d./7d + tara gum, followed by 1 tablet every 3 nights for 3 weeks, finally per week for 56d	Italy	-significantly reduced the Nugent score below the threshold of 7 after 28 days compared to placebo group	(156)
95 non-pregnant BV and/or VVC patients	RDBPCT - vginal 10^8^ to 10^10^ cfu each of *L*. *gasseri* LN40. *L*. *fermentum* LN99. *L*. *casei* subsp. *rhamnosus* LN113. *P*. *acidilactici* LN23 1 capsule/o.d./5 d	Sweden	-led to the vaginal adherence of lactobacilli, -fewer recurrences, -less malodorous discharge	(157)
160 non-pregnant Women who needed to rebalance/or restore their vaginal microbiota	RDBPCT - vaginal >10^5^ cfu/mL *L*. *fermentum* 57A. *L*. *plantarum* 57B. *L*. *gasseri* 57C 1 capsule/o.d./d	Poland	-significant decrease in both vaginal pH -a significant increase in the abundance of lactobacilli spp. between visits	(149)
24 non pregnant premenopausal women BV	RDBPCT- vaginal A phase 2 RP study 2 × 10^9^ cfu/dose of *L. crispatus* CTV-05 (LACTIN-V) 1 capsule/o.d./5d then once weekly for 2 weeks	USA	-increased adherence by *L. crispatus* -Adverse effects AEs were evenly distributed between the LACTIN-V and placebo group	(146)
65 non-pregnant HIV-infected women with BV (18-45 yrs)	RDBPCT – oral 2 × 10^9^ cfu/dose of *L. rhamnosus* GR-1 *L. reuteri* RC-14 1 capsule/t.d./6 months	Tanzania	- No enhanced cure rate of BV among women with HIV treated with adjuvant probiotics to metronidazole treatment -women with an intermediate vaginal flora, probiotics tended to increase the probability of a normal vaginal flora	(158)
Non-pregnant 39 women with BV	PR, exploratory pilot study – vaginal *L. rhamnosus* DSM 14870 *L. gasseri* DSM 14869 1 × 10^8^ CFU 1 capsule/o.d./30d followed by once weekly for 190 days	South Africa	- increased adherence of the probiotics but probiotic did not improve BV cure rates or alleviate recurrence	(159)
Non-pregnant 50 women with BV (18-55 yrs)	Postbiotic gel *Lacticaseibacillus paracasei* ProSci-92 *Lacticaseibacillus rhamnosus* ProSci-109 1 × 10^6^ cfu/mL Daily for 7 days 1 capsule/o.d./7d	China	-relative abundance of vaginal lactobacilli increased compared to baseline -reduced *Gardnerella*, *Prevotella*, and *Atopobium*	(160)
Non-pregnant 310 BV patients	RDBPCT - oral 100 g probiotics/t.d/week) or 300 mg clindamycin/t.d/week	Iran	-BV recurrence in 10 and 9 in probiotics and clindamycin respectively -Ph decreased in 132 in probiotic and 105 in clindamycin -140 and 142 in probiotics and clindamycin respective had cure. -PTB in 12 and 9 probiotic and clindamycin subjects respectively -PPROM in 9 and 5 probiotic and clindamycin respectively	(161)

RPCT, randomized placebo-controlled trial; PR, partially randomized; RDBPCT, randomized double-blind placebo-controlled trial; o.d, once daily; t.d., twice daily; d, days.

However, other studies have revealed that probiotics do not offer a cure for BV and do not significantly impact pregnancy outcomes when compared to the placebo group (152, 154, 155). The uncertainty regarding their effectiveness arises from the wide variability in routes of administration, dosages, and probiotic choices, making it challenging to draw direct comparisons across studies. For instance, despite efforts to target specific effects and induce compositional changes in the cervicovaginal environment through oral administration, multiple studies have reported no significant differences between the placebo and probiotic-treated groups (**Table 2**) (154, 155, 161). These collective findings suggest that the oral route of administration may not be effective in achieving the desired effects in the cervicovaginal environment and, consequently, a protective effect against sPTB. The effectiveness of the vaginal route of administration, while appearing promising, is also challenged by the lack of uniformity surrounding its application.

## 2 Conclusion and future perspective

sPTB is a complex syndrome influenced by various factors. Research efforts have increasingly centered on investigating the role of the cervicovaginal microbiota in adverse pregnancy outcomes. While a definitive causal link is yet to be established, changes in microbial composition, characterized by a decrease in lactobacilli and an increase in opportunistic pathogens such as *Gardnerella*, *Enterobacter*, *Escherichia coli*, *Candida*, and *Prevotella*, have been associated with sPTB. The microbiota dynamics encompassing the production of lactic acid as well as bacteriocins, can have antagonistic effects critical for maintaining cervicovaginal microbiota balance that have profound influence on pregnancy outcome.

Moreover, the utilization of SCFAs and biosurfactants by cervicovaginal microbiota to adherence to epithelial surfaces, along with the presence of their proteolytic and glycohydrolase enzymes, may compromise the integrity of the epithelial. This could potentially impact the microbiota composition, increase susceptibility to infections, and ultimately influence the risk of sPTB. Nevertheless, our understanding of the production and function of these metabolites is restricted due to a paucity of comprehensive research.

Existing reports on cervicovaginal microbial composition and structure often rely on isolated time points rather than longitudinal studies, potentially missing crucial changes throughout pregnancy. Besides, the precise role of certain pathogens, even when present in low abundance, in triggering adverse pregnancy outcomes remains obscured. Therefore, conducting detailed microbial profiling throughout the gestational period is imperative to elucidate the impact of alterations in opportunistic pathogens and lactobacilli during gestation on pregnancy outcomes.

Traditionally, antibiotics have been employed to manage conditions like BV, AV, and other cervicovaginal infections. However, the use of antibiotics, with potential effects on lactobacilli, has been linked to high recurrence rates and chronic infections. Biotherapeutics such as probiotics have shown promise in reducing infections and relapses of BV and AV. Nevertheless, uncertainties persist regarding their effectiveness due to inadequate study power, lack of standardization in probiotic selection, dosage, administration routes, and study endpoints. Furthermore, the use of maternal synthetic preImplantation factor (sPIF) prophylaxis during pregnancy, which has shown promise in reducing bacterial lipopolysaccharide-induced preterm births by regulating exaggerated immune responses (162), may offer a viable option for managing, may provide a viable option for managing sPTB.

In conclusion, investigating the cervicovaginal microbiota and its interactions including host immunity during pregnancy has the potential to significantly enhance our understanding of sPTB and contribute to the development of effective prevention and treatment strategies.

## Author contributions

SO: Conceptualization, Data curation, Methodology, Visualization, Writing – original draft, Writing – review & editing. JM: Conceptualization, Methodology, Writing – review & editing. AK: Conceptualization, Funding acquisition, Writing – review & editing. PO: Funding acquisition, Supervision, Writing – review & editing. MT: Conceptualization, Funding acquisition, Supervision, Writing – review & editing. PV: Conceptualization, Funding acquisition, Writing – review & editing. RT: Conceptualization, Funding acquisition, Methodology, Supervision, Writing – review & editing. GO: Conceptualization, Funding acquisition, Methodology, Supervision, Writing – review & editing.

## Group member of the PRECISE Network

King’s College London (Laura A. Magee, Hiten Mistry, Marie-Laure Volvert, Lucilla Poston, Jane Sandall, Sophie Moore, Tatiana Salisbury); Aga Khan University, Nairobi (Grace Mwashigadi, Consolata Juma, Isaac Mwaniki, Alex Mugo, Joseph Mutunga, Moses Mukhanya, Onesmus Wanje, Marvin Ochieng, Emily Mwadime); Centro de Investigação de Saúde de Manhiça (Esperança Sevene, Corssino Tchavana, Salesio Macuacua, Anifa Vala, Helena Boene, Lazaro Quimice, Sonia Maculuve, Eusebio Macete, Inacio Mandomando, Carla Carillho); Donna Russell Consulting (Donna Russell); London School of Hygiene and Tropical Medicine (Hannah Blencowe, Veronique Filippi, Joy Lawn, Matt Silver, Joseph Waiswa, Ursula Gazeley); Midlands State University (Prestige Tatenda Makanga, Liberty Makacha, Reason Mlambo); MRC Unit The Gambia at LSHTM (Umberto D’Alessandro, Anna Roca, Hawanatu Jah Andrew M. Prentice, Melisa Martinez-Alvarez,Brahima Diallo, Abdul Sesay, Sambou Suso, Baboucarr Njie, Fatima Touray,Yahaya Idris, Fatoumata Kongira, Modou F.S. Ndure, Lawrence Gibba, Abdoulie Bah, Yorro Bah); University of Oxford (Alison Noble, Aris Papageorghiou, Rachel Craik); St George’s, University of London (Judith Cartwright; Guy Whitley, Sanjeev Krishna); University of British Colombia (Marianne Vidler, Jing (Larry) Li, Jeff Bone, Mai-Lei (Maggie) Woo Kinshella, Domena Tu, Ash Sandhu, Kelly Pickerill); Imperial College London (Ben Barratt).

## Glossary

**Table:**

Microbiome Microbiotaa Lactobacilli Microbiota Probiotics	a collection of all microbes, such as bacteria, viruses, fungi, and their associated genes, that are naturally on and in the living host (reasonably defined habitat). collection of microbes including bacteria, fungi, and viruses on and in the host. represents a group of bacteria that were classified as Lactobacillaceae until 2020 (163). diversity a measure of how many distinct species, and how evenly distributed they are in the community. Short-chain fatty acids—fatty acids with less than six carbon atoms that are produced by bacterial fermentation. live microbes that, when administered in adequate amounts, confer a health benefit to the host.
