# Corrigendum: Microbiota dynamics, metabolic and immune interactions in the cervicovaginal environment and their role in spontaneous preterm birth

**DOI:** 10.3389/fimmu.2024.1374545

**Published:** 2024-02-16

**Authors:** Stanley Onyango, Jia Dai Mi, Angela Koech, Patricia Okiro, Marleen Temmerman, Peter von Dadelszen, Rachel M. Tribe, Geoffrey Omuse, the PRECISE Network

**Affiliations:** ^1^ Department of Pathology, Aga Khan University, Nairobi, Kenya; ^2^ Centre of Excellence Women and Child Health, Aga Khan University, Nairobi, Kenya; ^3^ Faculty of Life Sciences and Medicine, Department of Women and Children’s Health, School of Life Course and Population Sciences, King’s College London, London, United Kingdom

**Keywords:** lactobacilli, preterm births, bacteriocins, lactic acid, pregnancy, probiotics

In the published article, there was an error in the author list, and the consortium **the PRECISE Network** was not credited with authorship. The corrected author list appears below.

Stanley Onyango^1,2*^, Jia Dai Mi^3^, Angela Koech^2^, Patricia Okiro^1^, Marleen Temmerman^2^, Peter von Dadelszen^3^, Rachel M Tribe^3^†, Geoffrey Omuse^1^†, ***and the PRECISE Network***

The authors apologize for this error and state that this does not change the scientific conclusions of the article in any way. The original article has been updated.

